# Development of aqueous two-phase systems-based approaches for the selective recovery of metalloproteases and phospholipases A_2_ toxins from *Crotalus molossus nigrescens* venom

**DOI:** 10.1186/s40643-021-00487-y

**Published:** 2021-12-28

**Authors:** Daniela Enriquez-Ochoa, David Meléndez-Martínez, José Manuel Aguilar-Yáñez, Cuauhtemoc Licona-Cassani, Karla Mayolo-Deloisa

**Affiliations:** 1 Tecnologico de Monterrey, School of Engineering and Sciences, Centro de Biotecnología-FEMSA, Av. Eugenio Garza Sada 2501 Sur, 64849 Monterrey, NL Mexico; 2 Tecnologico de Monterrey The Institute for Obesity Research, Av. Eugenio Garza Sada 2501 Sur, 64849 Monterrey, NL Mexico

**Keywords:** Aqueous two-phase systems, Phospholipases A2, Metalloproteases, *Crotalus molossus nigrescens*, Venom, Recovery

## Abstract

Snake venoms are rich sources of proteins with potential biotechnological and pharmaceutical applications. Among them, metalloproteases (MPs) and phospholipases A2 (PLA_2_) are the most abundant. Their isolation involves a multistep chromatographic approach, which has proven to be effective, however implies high operating costs and long processing times. In this study, a cost-effective and simple method based on aqueous two-phase systems (ATPS) was developed to recover MPs and PLA_2_ from *Crotalus molossus nigrescens* venom. A system with PEG 400 g mol^−1^, volume ratio (V_R_) 1, tie line length (TLL) 25% w/w and pH 7 showed the best performance for PLA_2_ recovery. In systems with PEG 400 g mol^−1^, V_R_ 1, TLL 15% w/w, pH 7 and 1 and 3% w/w of NaCl, selective recovery of MP subtype P-III was achieved; whereas, in a system with PEG 400 g mol^−1^, V_R_ 1, TLL 25% w/w and pH 8.5, MP subtypes P-I and P-III were recovered. Due to their low costs, ethanol–salt systems were also evaluated, however, failed to differentially partition PLA_2_ and MPs. The use of ATPS could contribute to the simplification and cost reduction of protein isolation processes from snake venoms and other toxin fluids, as well as potentially aid their biochemical, proteomic and biological analyses.

**Graphic Abstract:**

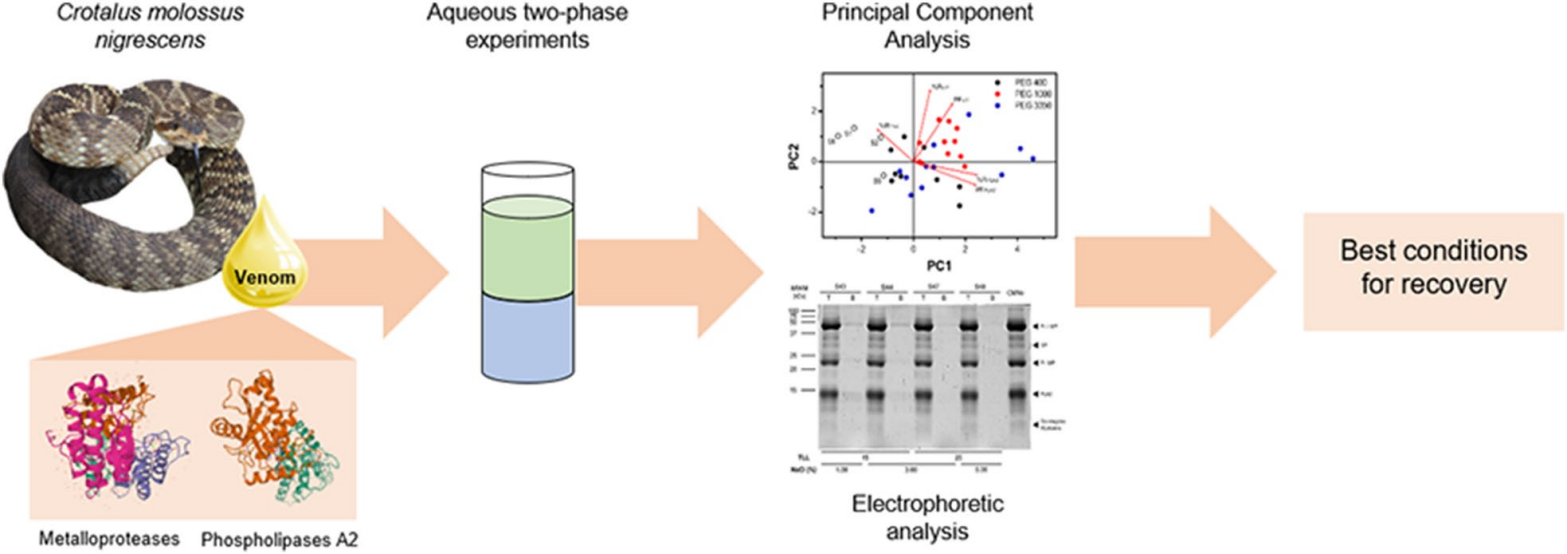

**Supplementary Information:**

The online version contains supplementary material available at 10.1186/s40643-021-00487-y.

## Introduction

Snake venoms are complex toxin cocktails comprising enzymatic and non-enzymatic proteins, such as phospholipases A2 (PLA_2_), metalloproteases (MPs), serine proteases (SPs), cysteine-rich secreted proteins, L-amino acid oxidases, C-type lectins, myotoxins, disintegrins, natriuretic peptides, hyaluronidases, nucleosidases and growth factors (Mackessy 2009). Among its components, PLA_2_ and MPs have been identified as the most abundant, playing a primary role in many deleterious effects of snake envenomation (Tasoulis and Isbister 2017). The study of these toxins is of growing interest for biotechnological and pharmaceutical applications, as they can be used for antivenom design, development of new drugs, and elucidation of the mechanism of action of venoms (De Marco Almeida et al. 2015; Gutiérrez et al. 2017; Laustsen 2018; Bermúdez-Méndez et al. 2018). A key aspect in the study of venom toxins is purity. Pure toxins are required for characterization, identification, and functional studies, and to avoid non-desired synergistic interactions among venom components (Xiong and Huang 2018).

Due to the high complexity of snake venoms, the isolation of PLA_2_ and MPs usually involves two or more chromatographic separations, including molecular exclusion, ion exchange, reverse phase and affinity (Serino-Silva et al. 2018; Ferreira et al. 2018; Simões-Silva et al. 2021). The use of a multistep chromatographic approach has proven to be effective, but it is costly, requires specialized equipment, and long operating times. New approaches are needed for the development of optimized and economic processes. Aqueous two-phase systems (ATPS) are an attractive alternative to increase purity while minimizing the number of chromatographic steps (Pereira et al. 2020). ATPS have demonstrated to be useful as a partial recovery first step in downstream processes since it allows to remove a large quantity of contaminants. For example, ATPS have been incorporated as pre-purification steps of different monoclonal antibodies from transgenic tobacco (Platis and Labrou 2009).

ATPS are formed when two immiscible aqueous solutions above a certain critical concentration are combined. The recovery and purification of proteins is commonly performed in polymer–polymer and polymer–salt systems due to their high water content and low interfacial tension. Additionally, polymers may have a stabilizing effect on the protein structure (Varadavenkatesan et al. 2021). As an inexpensive alternative to this type of systems, alcohol–salt ATPS have been efficiently used for the separation of various proteins, such as prolyl endopeptidase, elongation factor 1γ and green fluorescent protein (Lo et al. 2018; Oliveira Filho et al. 2020; Jiang et al. 2021). Alcohol–salt ATPS includes advantages such as easy constituent recovery and reutilization, high polarity, low toxicity and low viscosity. When using alcohol–salt ATPS, the compatibility of the protein with the alcohol-rich phase should be considered to avoid denaturation or inactivation (Rito-Palomares and Benavides 2017). ATPS have several advantages compared to conventional purification techniques, such as being relatively simple and inexpensive, easily operated and scaled up, capability of process integration, and providing a biocompatible environment to maintain biological activity (Pereira et al. 2020). While ATPS are widely used for recovering proteins from complex matrices, application for recovering proteins from venoms is uncommon. To our knowledge, only three other studies have reported the use of this technique for the isolation of PLA_2_ and MPs from snake venoms (Gómez et al. 2012; Da Silva et al. 2015; Gomez et al. 2016). The large variation of venoms composition and activities, both at intra and interspecies levels, makes necessary the establishment of recovery conditions based on particular snake species from a certain region. Currently, there are no reports on the recovery of proteins from *Crotalus molossus nigrescens* venom using ATPS. This snake has the largest distribution in Mexico among *Crotalus molossus* subspecies (Borja et al. 2018). Previous analyses have shown that *C. m. nigrescens* venom has proteases, PLA_2_, phosphodiesterase, deoxyribonuclease, fibrinogen coagulase, collagenase and fibrinolytic activities (Ramírez et al. 1990). Among *Crotalus* species venoms, *C. m. nigrescens* venom has shown to be one with strongest proteolytic activity and PLA_2_ activity over erythrocytes, as well as having a high MP content (Ramírez et al. 1990; Macias-Rodríguez et al. 2014; Borja et al. 2018; Roldán-Padrón et al. 2019). From this venom, only two toxins have been isolated using chromatographic methods, a 21.4 kDa P-I MP and a 75 kDa SP (Ramírez et al. 1990).

In this study, the recovery of MPs and PLA_2_ from *C. m. nigrescens* venom using ATPS (ethanol–potassium phosphate and polyethylene glycol–potassium phosphate) were explored. The effect of the volume ratio (V_R_; volume of the top phase divided by the volume of the bottom phase), tie line length (TLL; final mass concentration of phase components in the top and bottom phases), polyethylene glycol (PEG) molecular weight, pH and sodium chloride (NaCl) addition was investigated to optimize the recovery of both enzymes. This study will contribute to the establishment of a simple and cost-effective alternative method for the recovery of MPs and PLA_2_ from snake venoms and potentially facilitate their study and biotechnological and pharmaceutical applications.

## Materials and methods

### Snake venom

*C. m. nigrescens* venom samples were obtained from specimens maintained in captivity at Universidad Autónoma de Querétaro Herpetary (Av. de las Ciencias, Santa Rosa Jaureguí, Queretaro, 76230, Mexico) under permission of Dirección General de Vida Silvestre (Permit No: INE/CITES/DGVS-CR-IN-0619-QRO00). Venom extraction was performed as described by Meléndez-Martínez et al. (2014). After extraction, venom was pooled, lyophilized and stored at −20 °C until use. Prior to use, lyophilized *C. m. nigrescens* venom was solubilized in distilled water and centrifuged for 15 min at 20,400*g* using a Prism R centrifuge (Labnet, NJ, USA) to remove insoluble proteins and cellular debris.

## Partitioning by aqueous two-phase systems

PEG–potassium phosphate ATPS were constructed based on the binodal curves reported previously by Zaslavsky (1995). The composition of PEG–potassium phosphate systems is presented in Additional file 1: Table S1. The systems were prepared by weighing predetermined amounts of PEG nominal molecular weights of 400 (100% w/w solution), 1000 (40% w/w solution) and 3350 (40% w/w solution) g mol^−1^ (Sigma-Aldrich, St Louis, MO, USA), potassium phosphate buffer (K_2_HPO_4_–KH_2_PO_4_, ratio 18:7, pH 7–10, J.T. Baker, PA, USA) and water. Ethanol–potassium phosphate ATPS were constructed based on the binodal curve reported previously by Gómez-Loredo et al. (2014). The composition of ethanol–potassium phosphate systems is shown in Additional file 1: Table S2. The systems were prepared by weighing appropriate amounts of ethanol (96%, D.E.Q., NL, Mexico), potassium phosphate buffer (K_2_HPO_4_–KH_2_PO_4_, ratio 18:7, pH 7, J.T. Baker, PA, USA) and water. Following this, 0.2 g of venom solution at a total protein concentration of 5 mg/mL were added to each system to obtain a system total weight of 2 g. Systems were thoroughly mixed by gentle agitation for 15 min at 4 ºC. Complete phase separation was achieved by centrifuging at 11,200 *g* for 10 min at 4 ºC using a Prism R centrifuge (Labnet, NJ, USA). The final volume of each phase was determined visually in graduated tubes. Then, each phase was carefully separated with the aid of a micropipette for total protein, caseinolytic, PLA_2_ activity determination or SDS-PAGE analysis. All samples were analyzed against a blank system prepared with 0.2 g of water instead of venom solution. The top phase recovery percentage (%R) and purification factor (PF) were further calculated using the following equations:

1 $$\% {\text{R}} = \left( {{\text{U}}_{{{\text{top}}}} /{\text{U}}_{{{\text{loaded}}}} } \right) \, \times {1}00,$$

where U_top_ is the total enzymatic units of MPs or PLA_2_ or total protein in the top phase, and U_loaded_ is the total enzymatic units of MPs or PLA_2_ or total protein in 0.2 g of venom solution:

2 $${\text{PF}} = {\text{Act}}_{{{\text{top}}}} /{\text{Act}}_{{{\text{venom}}}} ,$$

where Act_top_ is the specific activity of MPs or PLA_2_ in the top phase, and Act_venom_ is the specific activity of MPs or PLA_2_ in 0.2 g of venom solution.

To study the recovery of MPs and PLA_2_ in PEG–potassium phosphate systems, the effect of several system parameters was evaluated. Initially, the effects of the PEG molecular weight (400, 1000 and 3350 g mol^−1^), TLL (15, 25, 35 and 45% w/w) and V_R_ (0.33, 1 and 3) were analyzed. Then, the effect of NaCl addition in MPs and PLA_2_ recovery was investigated in the systems with best separation performance (TLL 15 and 25% w/w, PEG 400 g mol^−1^ and V_R_ 0.33 and 1). Finally, improvement of the enzymes partition was attempted by varying the pH (7–10) of the systems.

In ethanol–potassium phosphate systems, the effect of V_R_ on MPs and PLA_2_ partition was investigated at 0.33, 1 and 3. All of the systems were constructed at TLL 40% w/w.

### Total protein quantification

Total protein concentration of *C. m. nigrescens* venom and ATPS phases were determined by the Bradford protein assay using bovine serum albumin (Bio-Rad Laboratories, CA, USA) as standard (Bradford 1976). Briefly, 10 µL of sample were mixed with 250 µL of Bradford reagent (Sigma-Aldrich, St Louis, MO, USA) and incubated 10 min. After, absorbance was measured at 595 nm using a Synergy HT microplate reader (Biotek, VT, USA).

### Caseinolytic activity determination

Protease activity was determined using casein (Lamesa S.A. de C.V., Gto, Mexico) as substrate according to the method of Das et al. (2013)*.* Appropriate amounts of *C. m. nigrescens* venom and ATPS phases were diluted in 100 µL of phosphate buffered saline (PBS) pH 7.4 (Sigma-Aldrich, MO, USA) and incubated with 200 µL of 1% (w/v) casein in 20 mM Tris–HCl buffer, pH 7.4, for 1 h at 37 °C. The reaction was stopped with cold 15% (w/v) trichloroacetic acid (TCA; J.T. Baker, PA, USA) and centrifuged for 15 min at 450 *g* using a Prism R centrifuge (Labnet, NJ, USA). Supernatant was collected and soluble digested protein was determined by ninhydrin-based protein assay using L-leucine (Sigma-Aldrich, MO, USA) as a standard (Starcher 2001). The units of protease enzymatic activity were defined as one mmol equivalent of L-leucine formed per minute per mL (Das et al. 2013).

### PLA_2_ activity determination

PLA_2_ activity was determined according to the protocol of Corrigan et al. (Corrigan et al. 1983) with modifications. An egg yolk was dissolved in 1 L of 0.9% (w/v) NaCl. Egg yolk solution (50 µL) was added to appropriate amounts of *C. m. nigrescens* venom and ATPS phases diluted in 200 µL of PBS pH 7.4. The reaction was incubated at 37 °C and turbidity was measured at 925 nm at 5 min and 15 min using a Synergy HT microplate reader (Biotek, VT, USA). The units of PLA_2_ enzymatic activity were defined as the difference of turbidity at 5 min and 15 min per mL per min.

### Effect of pH on enzymatic activity

The effect of pH on enzymatic stability was performed incubating the *C. m. nigrescens* venom at 37 °C for 15 min in 50 mM acetate buffer (pH 4–5.5), 50 mM phosphate buffer (pH 6–8) and 50 mM Tris–HCl buffer (8.5–10). After incubation, caseinolytic and PLA_2_ activities were determined as previously described.

### SDS-PAGE analysis

For SDS-PAGE analysis, enzymes were recovered from PEG and potassium phosphate phases using 3 kDa Amicon ultrafilters (Merck Millipore, MA, USA); whereas, enzyme recovery from the ethanol phase was performed using a SAVANT centrifugal evaporator (Thermo Fisher Scientific, MA, USA). The protein pattern from 10 µg of *C. m. nigrescens* venom and ATPS phases were observed in a 12% SDS-PAGE gel according to the method of Sambrook and Russell (Sambrook and Russell 2001) and stained with Coomassie colloidal stain (Bio-Rad Laboratories, CA, USA) (Dyballa and Metzger 2009), using Precision Plus Protein Dual Xtra (Bio-Rad Laboratories, CA, USA) as a molecular weight marker. Densitometric analysis was performed with SDS-PAGE and using ImageJ 1.8 software (U.S. National Institutes of Health, Bethesda, Maryland, USA). Densitometry was calculated using the following equation:

3 $${\text{Densitometry}} = \left( {{\text{PD}}_{{{\text{sample}}}} {\text{/PD}}_{{{\text{venom}}}} } \right) \times {\text{1}}00 ,$$

where PD_sample_ is the pixel density of the MPs or PLA_2_ bands in the selected ATPS phases, and PD_venom_ is the pixel density of the MPs or PLA_2_ bands in *C. m. nigrescens* venom.

### Data analysis

Results were expressed as mean ± standard error according to the number of experiments performed. Each dependent variable (%R_MP_, %R_PLA2_, %R total protein, PF_MP_, and PF_PLA2_) was analyzed through one-way ANOVA (*p* < 0.05). When ANOVA showed significative differences, Tukey post hoc test was performed. Data were analyzed using a multivariate statistical test, specifically, principal component analysis (PCA). PCA allowed data reduction, and graphical examination of independent variables effect over dependent variables. All experiments were carried out at least in triplicate. The statistical analyses were done in Minitab 18 (PA, USA) and plotted in Prism Graph Pad 6.

## Results and discussion

### Recovery of MPs and PLA_2_ in PEG–salt systems

To study the recovery of MPs and PLA_2_ in PEG–salt systems, different system parameters, including PEG molecular weight, V_R_, TLL, NaCl addition and pH were evaluated. It is important to remark that in these systems, enzyme activity recoveries higher than 100% were obtained (Tables 1, 2 and 3). This behavior has been frequently reported for several enzymes, including MPs and phospholipases, when they are recovered using ATPS (Cavalcanti et al. 2006; Babu et al. 2008; Porto et al. 2008; Karkaş and Önal 2012; Duque Jaramillo et al. 2013; Ketnawa et al. 2013; Nascimento et al. 2016; Vázquez-Villegas et al. 2018). PEG can alter the structure of the enzyme active sites, and consequently, might enhance its relative activity (Pancera et al. 2002; Babu et al. 2008; Porto et al. 2008; Karkaş and Önal 2012). Enzyme activation could also be attributed to the decrease or elimination of potential inhibitors in the phases during the partitioning process (Mayerhoff et al. 2004; Karkaş and Önal 2012; Nascimento et al. 2016). The presence of MPs inhibitors in vipers’ venom has been demonstrated in several reports (Segura et al. 2017; Leonardi et al. 2019; Giribaldi et al. 2020). Other possible mechanism for enzymes activation, particularly for MPs, includes stabilization against autolysis due to the presence of PEG (Chae et al. 2000). It has been reported that proteases from *C. m. nigrescens* venom are greatly unstable due to autolytic degradation (Ramírez et al. 1990). Also note that the enzymatic activity of PLA_2_ and MPs could not be quantified in the top phase of some PEG–salt systems, since the enzymatic activity was too low to be detected.

**Table 1:** Effect of PEG–potassium phosphate systems variables on the recovery of MPs and PLA_2_

System identifier	System parameters			Top phase recovery percentage (%R)			Top phase purification factor (PF)	
	PEG molecular weight (g mol^−1^)	V_R_	TLL (% w/w)	MPs	PLA_2_	Total protein	MPs	PLA_2_
S1	400	0.33	15	181.90 ± 23.31^b,c,d,e,f,g,h,i,j^	13.72 ± 3.47^n,o^	92.80 ± 25.87^a^	3.76 ± 1.55^ g,h,i,j,k,l,m^	0.48 ± 0.10^e^
S2			25	178.95 ± 15.41^b,c,d,e,f,g,h,i,j^	15.59 ± 0.31^n,o^	66.58 ± 12.37^a,b,c,d,e^	5.61 ± 0.98^e,f,g,h,i,j,k,l^	1.01 ± 0.19^e^
S3			35	161.32 ± 16.78^d,e,f,g,h,i,j,k^	15.72 ± 2.29^n,o^	72.14 ± 5.09^a,b,c^	5.58 ± 0.31^e,f,g,h,i,j,k,l^	1.10 ± 0.20^e^
S4			45	196.23 ± 0.85^b,c,d,e,f,g,h,i^	16.46 ± 3.37^ m,n,o^	63.70 ± 3.18^a,b,c,d,e,f,g^	7.74 ± 0.40^c,d,e,f^	13.20 ± 3.13^d,e^
S5		1.00	15	144.81 ± 27.25^e,f,g,h,i,j,k,l^	25.07 ± 2.62^ l,m,n,o^	88.75 ± 32.48^a^	1.71 ± 0.65^ m,n^	5.80 ± 1.46^e^
S6			25	131.60 ± 34.51^e,f,g,h,i,j,k,l^	13.47 ± 8.50^n,o^	47.93 ± 2.14^b,c,d,e,f,g,h,i,j^	2.33 ± 0.65^j,k,l,m,n^	5.11 ± 2.75^e^
S7			1	112.85 ± 68.77^ h,i,j,k,l^	79.20 ± 3.12^ k,l,m,n,o^	47.65 ± 3.07^b,c,d,e,f,g,h,i,j^	2.14 ± 1.38^ l,m,n^	31.38 ± 1.75^d,e^
S8			45	95.52 ± 57.87^i,j,k,l^	115.37 ± 22.92^i,j,k,l,m,n,o^	4.29 ± 3.48^ m,n^	2.58 ± 2.58^i,j,k,l,m,n^	479.90 ± 346.20^a,b^
S9		3.00	15	158.67 ± 10.37^d,e,f,g,h,i,j,k^	89.18 ± 4.93^j,k,l,m,n,o^	54.90 ± 2.20^b,c,d,e,f,g,h^	2.24 ± 0.22^ k,l,m,n^	25.08 ± 1.69^d,e^
S10			25	137.03 ± 21.98^e,f,g,h,i,j,k,l^	173.74 ± 11.75^ h,i,j,k^	47.03 ± 6.38^c,d,e,f,g,h,i,j^	2.07 ± 0.09^ l,m,n^	54.98 ± 8.48^d,e^
S11			35	155.22 ± 1.99^d,e,f,g,h,i,j,k,l^	336.73 ± 10.54^d,e^	39.83 ± 3.85^d,e,f,g,h,i,j,k,l^	3.01 ± 0.27^ h,i,j,k,l,m,n^	131.22 ± 14.59^c,d,e^
S12			45	161.32 ± 8.99^d,e,f,g,h,i,j,k^	474.34 ± 24.89^a,b,c^	41.94 ± 2.23^d,e,f,g,h,i,j,k,l^	2.89 ± 0.12^i,j,k,l,m,n^	171.12 ± 14.66^c,d,e^
S13	1000	0.33	15	177.77 ± 30.23^b,c,d,e,f,g,h,i,j^	125.35 ± 25.28^i,j,k,l,m,n^	48.88 ± 4.61^b,c,d,e,f,g,h,i^	7.24 ± 1.05^c,d,e,f,g^	101.62 ± 17.47^d,e^
S14			25	232.02 ± 21.22^a,b,c,d,e,f^	151.23 ± 33.00^i,j,k^	77.34 ± 5.71^a,b^	5.98 ± 0.11^e,f,g,h,i^	77.12 ± 14.50^d,e^
S15			35	275.38 ± 13.85^a,b^	205.21 ± 8.08^f,g,h,i,j^	67.91 ± 9.82^a,b,c,d^	8.94 ± 0.83^b,c,d,e^	135.50 ± 20.82^c,d,e^
S16			45	269.58 ± 7.49^a,b,c^	167.63 ± 11.26^ h,i,j,k^	65.02 ± 2.97^a,b,c,d,e,f^	10.40 ± 0.48^a,b,c^	128.85 ± 5.83^c,d,e^
S17		1.00	15	186.26 ± 34.06^b,c,d,e,f,g,h,i,j^	184.67 ± 5.86^ g,h,i,j,k^	47.20 ± 4.26^c,d,e,f,g,h,i,j^	4.31 ± 0.44^f,g,h,i,j,k,l,m^	88.50 ± 9.09^d,e^
S18			25	253.13 ± 49.13^a,b,c,d^	276.71 ± 28.48^e,f,g,h^	42.23 ± 6.61^c,d,e,f,g,h,i,j,k,l^	6.57 ± 0.35^d,e,f,g^	148.33 ± 9.33^c,d,e^
S19			35	234.02 ± 29.48^a,b,c,d,e^	299.16 ± 31.49^e,f,g^	45.18 ± 3.42^c,d,e,f,g,h,i,j,k^	5.73 ± 0.43^e,f,g,h,i,j,k^	147.51 ± 11.76^c,d,e^
S20			45	147.58 ± 35.78^e,f,g,h,i,j,k,l^	211.41 ± 72.20^f,g,h,i^	37.25 ± 8.58^e,f,g,h,i,j,k,l^	4.72 ± 0.12^f,g,h,i,j,k,l,m^	127.09 ± 21.61^c,d,e^
S21		3.00	15	227.59 ± 21.16^a,b,c,d,e,f,g^	422.05 ± 23.12^b,c,d^	29.12 ± 1.56^ h,i,j,k,l,m,n^	4.78 ± 0.32^f,g,h,i,j,k,l,m^	177.77 ± 7.00^c,d,e^
S22			25	229.25 ± 20.91^a,b,c,d,e,f,g^	319.55 ± 11.02^d,e,f^	29.50 ± 0.99^ h,i,j,k,l,m,n^	6.45 ± 0.39^d,e,f,g,h^	180.56 ± 0.99^c,d,e^
S23			35	331.84 ± 23.41^a^	282.01 ± 22.95^e,f,g,h^	30.32 ± 1.91^ h,i,j,k,l,m^	7.83 ± 0.46^b,c,d,e,f^	132.76 ± 5.44^c,d,e^
S24			45	232.78 ± 28.75^a,b,c,d,e^	207.86 ± 13.12^f,g,h,i^	17.94 ± 0.17^j,k,l,m,n^	8.82 ± 0.97^b,c,d,e^	157.76 ± 6.55^c,d,e^
S25	3350	0.33	15	84.14 ± 12.82^j,k,l^	76.58 ± 15.32^ k,l,m,n,o^	40.39 ± 2.61^d,e,f,g,h,i,j,k,l^	4.42 ± 0.24^f,g,h,i,j,k,l,m^	84.59 ± 21.20^d,e^
S26			25	71.20 ± 5.95^ k,l^	141.25 ± 1.62^i,j,k,l^	35.07 ± 2.35^f,g,h,i,j,k,l^	3.93 ± 0.07^ g,h,i,j,k,l,m^	157.91 ± 13.06^c,d,e^
S27			35	50.09 ± 18.70^ l^	117.09 ± 3.74^i,j,k,l,m,n,o^	46.37 ± 2.36^c,d,e,f,g,h,i,j^	2.76 ± 1.02^i,j,k,l,m,n^	132.69 ± 7.86^c,d,e^
S28			45	126.89 ± 16.39^f,g,h,i,j,k,l^	147.43 ± 4.98^i,j,k^	48.92 ± 1.41^b,c,d,e,f,g,h,i^	6.46 ± 0.74^d,e,f,g,h^	150.94 ± 6.52^c,d,e^
S29		1.00	15	125.59 ± 11.37^ g,h,i,j,k,l^	84.81 ± 3.30^ k,l,m,n,o^	34.05 ± 3.74^ g,h,i,j,k,l,m^	3.71 ± 0.17^ g,h,i,j,k,l,m^	51.28 ± 6.86^d,e^
S30			25	142.22 ± 14.86^e,f,g,h,i,j,k,l^	134.14 ± 4.89^i,j,k,l,m^	27.05 ± 0.63^ h,i,j,k,l,m,n^	5.83 ± 0.54^e,f,g,h,i,j^	110.18 ± 2.63^d,e^
S31			35	169.90 ± 32.55^c,d,e,f,g,h,i,j,k^	119.49 ± 7.61^i,j,k,l,m,n^	25.28 ± 2.63^ h,i,j,k,l,m,n^	8.71 ± 1.60^b,c,d,e^	121.80 ± 4.67^d,e^
S32			45	228.18 ± 58.38^a,b,c,d,e,f,g^	168.38 ± 14.32^ h,i,j,k^	18.64 ± 3.96^j,k,l,m,n^	13.69 ± 2.35^a^	212.18 ± 30.36^b,c^
S33		3.00	15	ND	ND	ND	ND	ND
S34			25	202.36 ± 19.32^b,c,d,e,f,g,h^	546.37 ± 6.58^a^	21.63 ± 1.89^i,j,k,l,m,n^	5.97 ± 0.17^e,f,g,h,i^	326.12 ± 18.81^b,c^
S35			35	228.30 ± 25.38^a,b,c,d,e,f,g^	538.82 ± 19.48^a,b^	15.15 ± 1.47^ k,l,m,n^	9.73 ± 1.78^b,c,d^	450.75 ± 34.06^b^
S36			45	211.20 ± 23.60^b,c,d,e,f,g,h^	373.49 ± 154.91^c,d,e^	14.24 ± 2.94^ l,m,n^	11.36 ± 1.77^a,b^	676.37 ± 104.65^a^

When the enzymatic activity was too low to be quantified, non-detected (ND) is indicated. Values in the same column with different letters are significantly different (*p* < 0.05)

**Table 2:** NaCl concentration effect on the recovery of MPs and PLA_2_ in PEG–potassium phosphate systems

System identifier	System parameters			Top phase recovery percentage (%R)			Top phase purification factor (PF)	
	V_R_	TLL (% w/w)	NaCl (%w/w)	MPs	PLA_**2**_	Total protein	MPs	PLA_**2**_
S37	0.33	15	1	53.83 ± 16.68^a,b^	180.54 ± 6.23^a^	50.37 ± 2.76^a^	2.14 ± 0.67^a,b^	143.72 ± 3.12^a,b^
S38			3	53.19 ± 15.25^a,b^	141.25 ± 5.64^a,b^	40.17 ± 2.11^b^	2.70 ± 0.85^a,b^	141.64 ± 10.62^a,b,c^
S39			5	46.25 ± 9.87^a,b,c,d^	55.78 ± 2.74^b^	30.60 ± 4.16^c,d^	2.65 ± 1.07^a,b^	59.43 ± 20.84^e,f^
S40		25	1	45.40 ± 6.39^a,b,c,d^	126.91 ± 1.56^a,b^	43.26 ± 2.94^b^	2.09 ± 0.23^a,b^	118.66 ± 9.52^a,b,c,d,e^
S41			3	51.24 ± 9.42^a,b,c^	141.88 ± 3.07^a,b^	40.42 ± 0.66^b^	2.54 ± 0.49^a,b^	140.51 ± 4.11^a,b,c,d^
S42			5	56.43 ± 1.95^a,b^	66.73 ± 4.40^b^	33.12 ± 1.74^c^	3.41 ± 3.41^a,b^	81.40 ± 8.71^d,e^
S43	1.00	15	1	ND	113.16 ± 56.77^a,b^	27.81 ± 1.23^c,d,e^	ND	85.00 ± 42.67^b,c,d,e^
S44			3	12.52 ± 7.96^c,d,e^	106.64 ± 53.36^a,b^	25.32 ± 1.10^d,e,f^	0.52 ± 0.33^a,b^	143.27 ± 13.39^a,b^
S45			5	66.55 ± 10.70^a^	121.23 ± 3.51^a,b^	19.58 ± 0.41 ^f,g^	3.79 ± 0.66^a^	137.83 ± 6.12^a,b,c,d^
S46		25	1	18.84 ± 131.67^b,c,d,e^	131.67 ± 16.14^a,b^	23.51 ± 1.06^e,f,g^	0.81 ± 0.64^a,b^	0.67 ± 0.00 ^f^
S47			3	9.73 ± 5.94^d,e^	100.81 ± 51.67^a,b^	24.58 ± 0.51^d,e,f^	0.42 ± 0.26^a,b^	83.39 ± 42.06^c,d,e^
S48			5	26.85 ± 20.26^a,b,c,d,e^	123.20 ± 6.50^a,b^	17.19 ± 1.04 h	2.30 ± 1.33^a,b^	154.71 ± 12.39^a^

When the enzymatic activity was too low to be quantified, non-detected (ND) is indicated. Values in the same column with different letters are significantly different (*p* < 0.05)

**Table 3:** Effect of pH on the recovery of MPs and PLA_2_ in PEG–salt systems

System identifier	System parameters			Top phase recovery percentage (%R)			Top phase purification factor (PF)	
	PEG molecular weight (g mol^−1^)	TLL (% w/w)	pH	MPs	PLA_2_	Total protein	MPs	PLA_2_
S49	0.33	15	7.5	273.49 ± 26.23^d,e^	31.18 ± 7.68^c,d^	93.22 ± 3.9^a,b^	5.89 ± 0.66^a,b,c,d,e,f,g^	6.83 ± 1.83^b,c^
S50			8.0	189.54 ± 21.07^e,f,g,h^	5.61 ± 2.86^e,f^	100.00 ± 3.72^a^	3.73 ± 0.50^d,e,f,g^	1.11 ± 0.58^c^
S51			8.5	180.78 ± 6.33^e,f,g,h^	1.87 ± 3.74^e,f^	62.15 ± 9.33^b,c,d^	6.06 ± 0.82^a,b,c,d,e,f^	1.06 ± 0.53^c^
S52			9.0	222.20 ± 14.62^e,f,g,h^	3.74 ± 2.03^e,f^	49.02 ± 2.72^d^	9.11 ± 0.75^a^	1.06 ± 0.86^c^
S53			9.5	378.24 ± 0.94^b,c,d^	ND	100.00 ± 6.32^a^	8.62 ± 0.20^a,b^	ND
S54			10.0	257.61 ± 15.97^d,e,f^	1.06 ± 0.53^e,f^	100.00 ± 8.32^a^	4.05 ± 0.11^d,e,f,g^	0.16 ± 0.08^c^
S55		25	7.5	277.70 ± 29.78^c,d,e^	48.33 ± 14.19^c^	82.79 ± 2.48^b,c^	6.69 ± 0.63^a,b,c,d,e^	11.70 ± 3.41^b^
S56			8.0	152.50 ± 5.45^e,f,g,h^	ND	100.00 ± 10.41^a^	2.82 ± 0.15^f,g,h^	ND
S57			8.5	126.93 ± 4.62^ g,h^	2.49 ± 0.31^e,f^	55.47 ± 11.18^c,d^	5.05 ± 1.17^c,d,e,f,g^	1.03 ± 0.34^c^
S58			9.0	131.34 ± 60.01^f,g,h^	ND	57.87 ± 8.18^c,d^	5.27 ± 2.59^b,c,d,e,f,g^	ND
S59			9.5	402.92 ± 19.49^a,b,c^	ND	100.00 ± 3.69^a^	6.88 ± 0.25^a,b,c,d^	ND
S60			10.0	213.77 ± 7.67^e,f,g,h^	ND	100.00 ± 10.76^a^	3.41 ± 0.34^e,f,g,h^	ND
S61	1	15	7.5	421.10 ± 52.34^a,b^	362.89 ± 4.50^b^	46.45 ± 3.45^d^	8.22 ± 0.68^a,b,c^	71.91 ± 5.92^a^
S62			8.0	219.28 ± 37.42^e,f,g,h^	10.29 ± 3.14^e,f^	87.40 ± 31.13^a,b,c^	2.89 ± 1.16^f,g,h^	1.49 ± 0.82^c^
S63			8.5	136.16 ± 8.32^f,g,h^	3.74 ± 3.06^e,f^	37.12 ± 3.47^d^	3.70 ± 0.26^d,e,f,g^	0.70 ± 0.70^c^
S64			9.0	172.32 ± 80.27^e,f,g,h^	14.73 ± 12.03^d,e,f^	49.62 ± 4.79^d^	3.19 ± 1.51^f,g,h^	1.92 ± 1.92^c^
S65			9.5	508.93 ± 25.41^a^	8.42 ± 6.87^e,f^	100.55 ± 2.85^a^	5.06 ± 0.12^c,d,e,f,g^	0.54 ± 0.54^c^
S66			10.0	239.52 ± 5.12^e,f,g^	ND	100.00 ± 9.10^a^	2.49 ± 0.16^ g,h^	ND
S67		25	7.5	457.04 ± 67.24^a,b^	386.90 ± 2.99^a^	47.49 ± 3.37^d^	8.14 ± 1.37^a,b,c^	68.65 ± 5.36^a^
S68			8.0	196.51 ± 21.30^e,f,g,h^	19.21 ± 6.54^d,e^	49.48 ± 1.75^d^	3.60 ± 0.28^d,e,f,g^	1.49 ± 1.17^c^
S69			8.5	131.16 ± 19.35^f,g,h^	5.14 ± 3.06^e,f^	32.00 ± 1.60^d,e^	3.75 ± 0.62^d,e,f,g^	1.02 ± 1.02^c^
S70			9.0	106.36 ± 74.35^ h^	11.55 ± 9.43^e,f^	44.86 ± 8.57^d^	2.76 ± 2.16^f,g,h^	2.30 ± 2.30^c^
S71			9.5	ND	ND	ND	ND	ND
S72			10.0	275.38 ± 15.76^c,d,e^	ND	100.00 ± 12.35^a^	3.08 ± 0.48^f,g,h^	ND

When the enzymatic activity was too low to be quantified, non-detected (ND) is indicated. Values in the same column with different letters are significantly different (*p* < 0.05)

#### Effect of PEG molecular weight, VR and TLL on the recovery of PLA_2_ and MPs in PEG–potassium phosphate systems

The influence of PEG molecular weight on MPs and PLA_2_ recovery was analyzed at three different values (400, 1000 and 3350 g mol^−1^). Results showed a similar behavior for most of the systems. The %R and PF of PLA_2_ and MPs increased when PEG molecular weight was changed from 400 to 1000 g mol^−1^ (Table 1). However, when PEG molecular weight was increased from 1000 to 3350 g mol^−1^, an opposite effect was observed; the %R and PF of PLA_2_ and MPs decreased (Table 1). This behavior suggests that several factors are involved in the recovery of both enzymes. An increase in PEG molecular weight reduces the free volume available in the PEG-rich phase for proteins (Yavari et al. 2013). This could explain the behavior of both enzymes when PEG molecular weight varied from 1000 to 3350 g mol^−1^. On the other hand, the increase in PLA_2_ and MPs recovery observed when PEG molecular weight was changed from 400 to 1000 g mol^−1^ could be attributed to an interaction between the enzymes and the polymer. As PEG molecular weight increases, the ratio of hydrophilic groups to hydrophobic area is reduced, occasioning a rise in hydrophobicity (Rito-Palomares and Benavides 2017). Therefore, if a protein has hydrophobic affinity, its partition will be enhanced towards the PEG-rich phase. Since PLA_2_ and MPs recovery is enhanced to the top phase when PEG molecular weight increases, it can be assumed that these enzymes have certain hydrophobic affinity.

The influence of the V_R_ was analyzed at three different values (0.33, 1 and 3). In most of the systems, the %R of both enzymes was greater at a V_R_ of 3 (Table 1). An increment of V_R_ implies an increase of the available free volume in the top phase, which overcomes saturation problems and promotes partition of the molecules towards the top phase (Benavides and Rito-Palomares 2008; Gómez-Loredo et al. 2014). A similar effect was observed by Gomez et al. (2016). As seen in Table 1, V_R_ modification had a different effect on the purity of each enzyme. At PEG molecular weights of 400 and 1000 g mol^−1^, the PF of MPs was enhanced when the V_R_ was reduced to 0.33. While at a PEG molecular weight of 3350 g mol^−1^, the PF of MPs increased as the V_R_ also increased. The PF of PLA_2_ reached its highest values when the V_R_ was raised to 3. This behavior could be associated to the affinity of MPs, PLA_2_ and contaminant proteins towards the top phase and the free volume available in both phases (Benavides and Rito-Palomares 2008). *C. m. nigrescens* venom comprised multiple proteins and components, and their partition behavior in the system affects the recovery and purity of MPs and PLA_2_.

Lastly, the effect of the TLL was analyzed. The effect of this parameter on the %R and PF of MPs and PLA_2_ varied according to the V_R_ and PEG molecular weight (Table 1). The free available volume in the system depends on the interaction of several factors including the V_R_, PEG molecular weight and TLL. It has been reported that free volume in the bottom phase decreases as the TLL increases, and in consequence the migration of proteins is promoted to the top phase (Benavides and Rito-Palomares 2004). Due to the relative affinity of both enzymes to the top phase, it was expected that at lower PEG molecular weights, higher V_R_ and TLL, higher recoveries would be obtained. However, results showed that this was not always the case, probably due to migration of other components and proteins present in the venom towards the top phase. As a consequence, the amount of MPs and PLA_2_ in this phase was reduced, and their recovery and purity was negatively affected (Aguilar et al. 2006; Mayolo-Deloisa et al. 2009). Note that in some of the systems, the PF remained relatively constant even when the TLL was modified. This could be attributed to compensation of the negative effect of contaminant proteins migrating to the top phase by a higher enzyme activity increase (Aguilar et al. 2006).

**Fig. 1:**
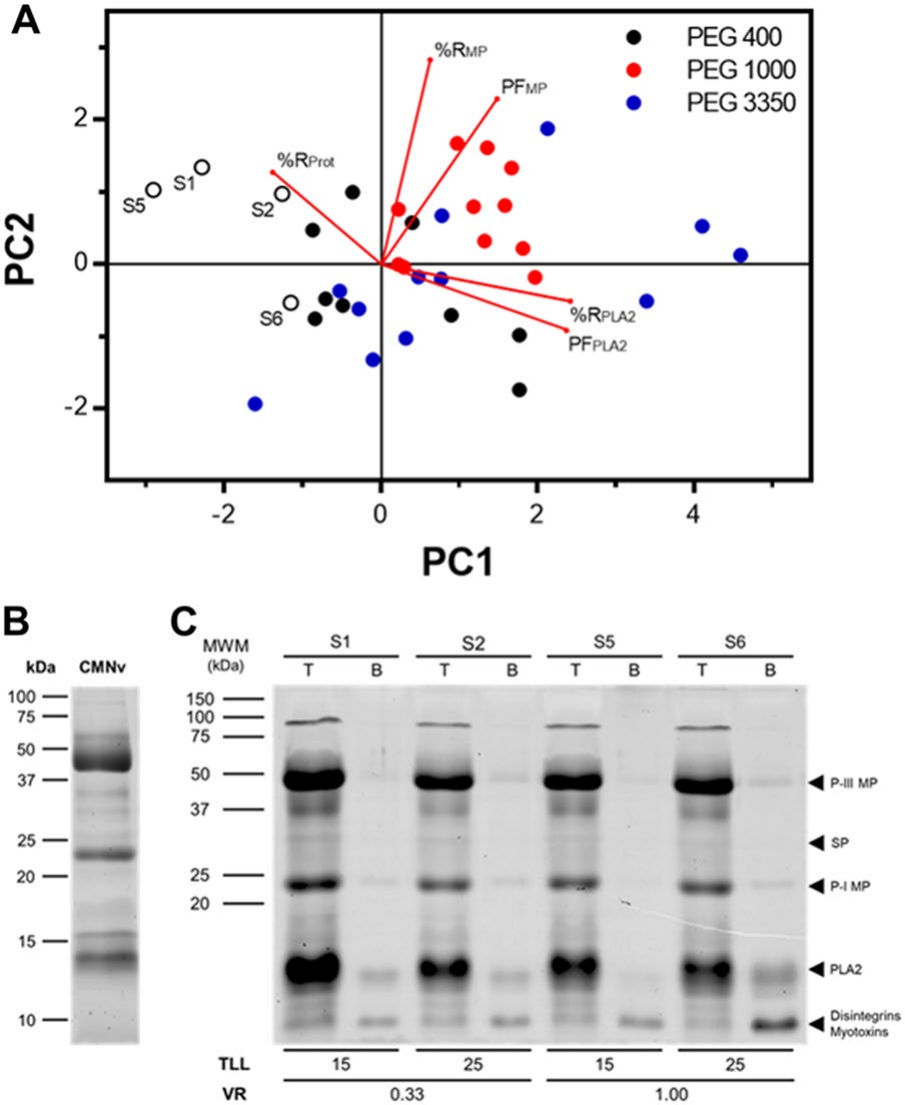
Effect of PEG–potassium phosphate systems’ variables on the recovery of MPs and PLA_2_. **A** Principal component analysis (PCA) of the recovery of MPs and PLA_2_ in systems with a PEG molecular weight of 400, 1000 and 3350 g mol^−1^, TLL 15, 25, 35 and 45% w/w and V_R_ 0.33, 1 and 3. Clear circles (○) denote the selected systems with the best partition characteristics. **B** SDS-PAGE of 15 μg of *C. m. nigrescens* venom (CMNv) and molecular weight marker (MWM). **C** SDS-PAGE of top and bottom phases (T and B, respectively) of selected ATPS from PCA analysis

The cumulative effects of PEG molecular weight, V_R_ and TLL on either PLA_2_ or MPs recovery and purity were analyzed using PCA (Fig. 1A). The PCA showed that the best systems to recover MPs in the top phase were found in the upper-right quadrant, as this zone has a higher recovery of total protein and MPs, and PF of MPs, whereas the systems observed in the lower-right quadrant had the best conditions to recover PLA_2_ recovery in the top phase. The explained variance of the dataset was 81.9%, suggesting a true tendency. The PCA suggested that the systems S1, S2, S5 and S6 were the most differentiated, with higher capacity to retain MPs in the top phase, partitioning the PLA_2_ to the bottom phase (Fig. 1C). In these systems, there was a high activity of MPs and low PLA_2_ activity in the top phase. Since enzymatic activity was affected by the phase components and this could lead to bias in the interpretation of separation performance, an electrophoretic and densitometric analysis of systems S1, S2, S5, and S6 was performed (Additional file 2: Figure S1A and C). The results showed that in all of the systems, PLA_2_ (14 kDa band) migrated to the bottom phase (Mackessy 2009). Interestingly, disintegrins and myotoxins (10 kDa band) were also observed in this phase, evidencing selective partition of these proteins. Notice that MPs are also present in the bottom phase, however in a lower amount than PLA_2_. Since system S6 showed a higher intensity of the band corresponding to PLA_2_ in the bottom phase, it was selected as the best system to recover this enzyme from the crude venom.

#### Effect of NaCl addition to PEG–potassium phosphate systems on the recovery of MPs and PLA_2_

In most cases, the addition of neutral salts to ATPS allows to modify the partition of proteins towards one of the phases (Amid et al. 2012). Therefore, to optimize the recovery of PLA_2_ and MPs, the addition of NaCl at different concentrations was investigated using systems S1, S2, S5 and S6 (PEG 400 g mol^−1^, TLL 15 and 25% w/w and V_R_ 0.33 and 1). According to the results (Table 2), when NaCl was added to the systems, the %R and PF of MPs decreased. An opposite effect was observed for PLA_2_; the top phase %R and PF increased by several orders of magnitude when NaCl was added. The highest R% and PF of PLA_2_ were achieved at a V_R_ 0.33, TLL 15% w/w and 1% w/w NaCl. The lowest R% and PF of MPs were obtained at a V_R_ of 1, TLL 15% w/w and 1% w/w NaCl.

The presence of NaCl may be affecting the recovery of PLA_2_ and MPs due to hydrophobic interactions (Rosa et al. 2007). The addition of NaCl produces an increase of the amount of water in the bottom phase required for the solvation of salt ions. As a result, the hydrophobicity difference between the two phases increases, promoting the migration of more hydrophobic proteins to the top phase (Rosa et al. 2007). Since NaCl addition to the systems decreased the recovery of MPs on the top phase, it is presumable that the hydrophobicity of PLA_2_ is higher than those of MPs. However, Farruggia et al. (Farruggia et al. 2004) suggested another possible mechanism, in which polymer excluded volume is the main factor driving protein partition in ATPS at high salt concentration. According to the authors, in systems with a salt concentration higher than 0.3 M a loss of structured water around the PEG molecule is induced (Farruggia et al. 2004). The loss of water structure involves a reduction of the polymer molecule specific volume, resulting in an increase in the volume available for the protein to migrate to the top phase (Reh et al. 2007).

The addition of NaCl also generates an electric potential difference between the phases which can drive the migration of proteins towards one phase depending on their charge. Chloride ions are water structure breakers and partition predominantly to the PEG-rich phase (Glyk et al. 2016). Thus, the PEG-rich phase becomes more negative when NaCl is added to the system and consequently, positively charged proteins migration is enhanced towards this phase (Andrews et al. 2005; Rosa et al. 2007). The isoelectric point (pI) of PLA_2_ in snake venom secretion is highly variable; the same venom contains both acidic and basic isoforms (Tonello and Rigoni 2017). A similar case occurs for MPs, since diverse subtypes and isoforms with a wide range of pIs are present in snake venoms (Calvete et al. 2009; Georgieva et al. 2010; Markland and Swenson 2013). When NaCl was added to the systems at pH 7 (system pH), PLA_2_ recovery was enhanced and MPs recovery diminished in the top phase. Therefore, based on this behavior, it can be assumed that the isoforms present in the venom are mostly basic and acidic for PLA_2_ and MPs, respectively.

**Fig. 2:**
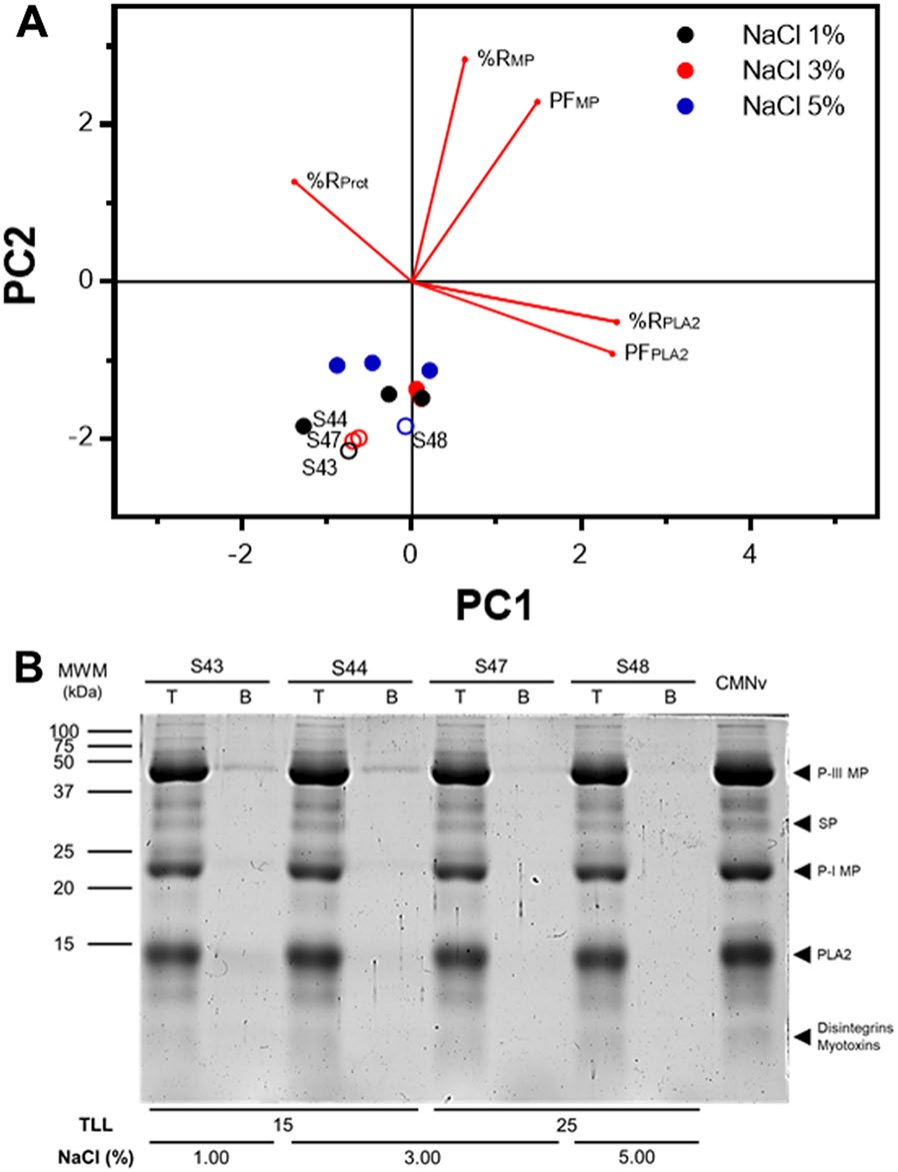
NaCl concentration effect on the recovery of MPs and PLA_2_ in PEG–potassium phosphate systems. **A** Principal component analysis (PCA) analysis of the recovery metalloproteases (MPs) and phospholipases A2 (PLA_2_) in selected systems at different NaCl concentrations (1, 3 and 5% w/w). Clear circles (○) denote the selected ATPS with the best partition characteristics. **B** SDS-PAGE of top and bottom phases (T and B, respectively) of selected ATPS from PCA analysis, 15 μg of *C. m. nigrescens* venom (CMNv) and molecular weight marker (MWM)

The PCA demonstrated that NaCl addition altered partition behavior of the enzymes, favoring partition of PLA_2_ to the top phase (Fig. 2A). Among the evaluated systems, S43, S44, S47, and S48 were the most differentiated with higher partitioning capacity of MPs and PLA_2_. Further electrophoretic analysis of these systems demonstrated that most PLA_2_ and MPs remained in the top phase of the systems, while the MP subtype P-III (46 kDa) was selectively partitioned to the bottom phase (Fig. 2B) (Mackessy 2009). The poor partition of MP P-III to the bottom phase in systems S43 and S44 (Additional file 2: figure S1B) does not explain the low activity of MPs observed in the top phase of these systems (Table 2), as this MP subtype confers major protease activity in *C. m. nigrescens* venom (Roldán-Padrón et al. 2019). This suggests that NaCl addition provided an unsuitable environment in the top phase for MPs activity and thus, could not be quantified. Since systems S43 and S44 showed a higher intensity of the band corresponding to MP P-III in the bottom phase, they were selected as the most suitable for recovering this enzyme.

### Effect of pH on the recovery of MPs and PLA_2_

An important parameter that affects protein partition is the pH of the system. The pH alters proteins superficial charge, which causes a variation in their migration towards certain phase. The influence of pH on the recovery of MPs and PLA_2_ was evaluated using systems S1, S2, S5 and S6 (PEG 400 g mol^−1^, TLL 15 and 25% w/w and V_R_ 0.33 and 1). Results showed that in a range from 8 to 10, both the %R and PF of MPs were superior from those obtained for PLA_2_ (Table 3). This behavior could be attributed to the influence of pH on electrochemical interactions. PEG has a positive dipolar momentum due to its terminal hydroxyl groups (Benavides and Rito-Palomares 2008). Thus, if the system pH is modified to values above the protein pI, enhanced affinity between PEG and the negatively charged protein is induced (Benavides and Rito-Palomares 2008; Asenjo and Andrews 2011; Mehrnoush et al. 2012; Rito-Palomares and Benavides 2017). Previous results suggested that MPs isoforms in the venom are mostly acidic (see 3.1.2. Effect of NaCl addition upon the partition of MPs and PLA_2_). Therefore, at a pH range of 8 to 10, MPs are negatively charged, and their partition is enhanced towards the top phase. The recovery and purity of MPs and PLA_2_ could also be influenced by the different protein families and isoforms in the venom, which depending on their charge will migrate preferentially towards one phase at the different evaluated pH values.

**Fig. 3:**
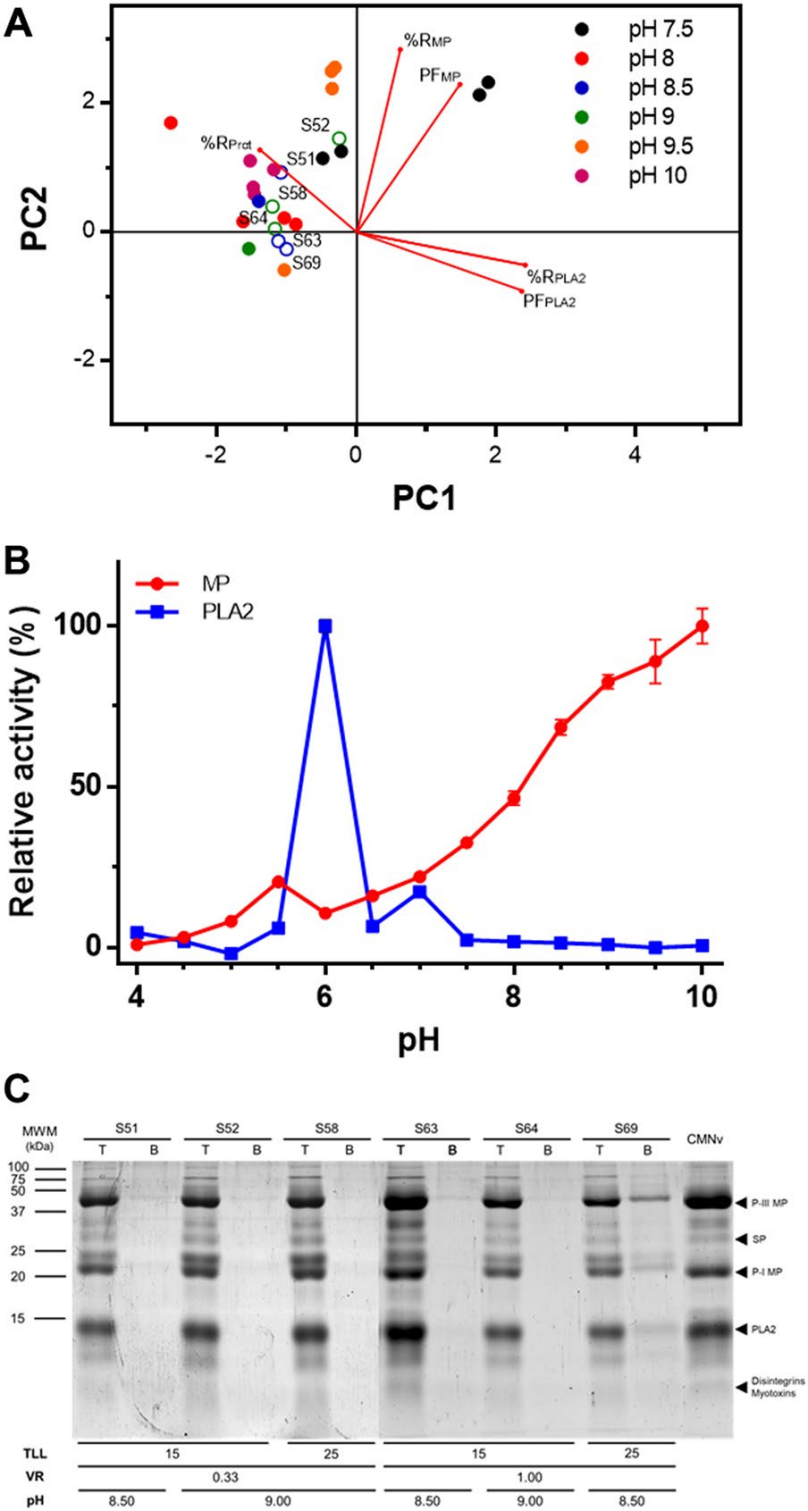
Effect of pH on the recovery of MPs and PLA_2_ in PEG–salt systems. **A** Principal component analysis (PCA) analysis of the recovery of MPs and PLA_2_ in selected systems at different pH values (7.5–10). Clear circles (○) denote the selected ATPS with the best partition characteristics. **B** Effect of pH on MPs and PLA_2_ activities. **C** SDS-PAGE of top and bottom phases (T and B, respectively) of selected ATPS from PCA analysis, 15 μg of *C. m. nigrescens* venom (CMNv) and molecular weight marker (MWM)

In this case, the PCA did not show a clear pattern of recovery for both enzymes (Fig. 3A). This behavior suggests that probably enzymatic activity was altered by pH variation and not by the partition of both enzymes and contaminant proteins to either of the phases. To get a better insight between the enzymatic activity and pH variation, PLA_2_ and MPs activities were evaluated at different pH values (Fig. 3B). Results showed that PLA_2_ has a higher activity at a neutral pH, while MPs at an alkaline pH. Since higher enzymatic activity due to pH variation could have caused a misleading increase of %R and PF for both enzymes, systems with higher partitioning capacity were chosen based on their total protein %R. In most of the systems, total protein %R suggested partition absence (Table 3). Therefore, those that had lower values of total protein %R were chosen to perform an electrophoretic analysis of partition (S51, S52, S58, S63, S64, and S69) (Fig. 3C). Among the evaluated systems, S69 showed the highest partitioning capacity. In the bottom phase of this system, three bands (46, 24 and 21 kDa) corresponding to different subtypes of MPs were observed along with a lower content of PLA_2_ compared to *C. m. nigrescens* crude venom (Additional file 1: figure S1C) (Markland and Swenson 2013). These results suggest that this system is the most adequate to recover MPs.

### Recovery of MPs and PLA_2_ in ethanol–potassium phosphate systems

Alcohol–salt systems have been used to purify enzymes effectively (Ooi et al. 2009; Amid et al. 2012; Simental-Martínez et al. 2014). In this work, ethanol–potassium phosphate systems were used to recover MPs and PLA_2_. The results showed that the activity of PLA_2_ in the top phase was enhanced (Table 4). The greatest top phase %R of PLA_2_ was obtained at a V_R_ of 1 (519.89%). Previous studies have shown that PLA_2_ stability and activity are increased in the presence of organic solvents, including ethanol (D’auria et al. 1999; Bacha et al. 2011). This change possibly occurs due to an alteration of the amount of water available for enzyme solvation, which in consequence affects catalytic activity and hydration of the active site (Carrea and Riva 2000; Yang et al. 2004). On the other hand, MPs recovery in the top phase was negatively affected. Amid et al. (2012) reported that ethanol decreased significantly the activity of SP in comparison to other organic solvents, such as 1-propanol and 2-propanol. They concluded that the longer hydrophobic chain of propanol provided a milder environment for the enzyme. The partition behavior of both enzymes in this type of system is in agreement with previous results (see 3.1.2. Effect of NaCl addition upon MPs and PLA_2_ partition). In alcohol–salt systems, compounds with higher hydrophobicity tend to migrate to the alcohol-rich phase, while hydrophilic compounds are transferred to the salt-rich phase (Ma et al. 2013). Similarly, as in PEG–salt systems with NaCl, it seems that PLA_2_ has a higher hydrophobicity than MPs and contaminant proteins.

**Table 4:** Recovery of metalloproteases (MPs) and phospholipases A2 (PLA_2_) using ethanol–potassium phosphate systems

System identifier	System parameters		Top phase recovery percentage (%R)			Top phase purification factor (PF)	
	PEG molecular weight (g mol^−1^)	Ethanol concentration (% w/w)	MPs	PLA_2_	Total protein	MPs	PLA_2_
S73	0.33	8.5	20.49 ± 6.87^a^	128.28 ± 6.13^a^	39.36 ± 0.78^a^	1.18 ± 0.38 ^a^	75.54 ± 1.39 ^a^
S74	1	16	50.83 ± 8.94^b^	519.89 ± 22.77^a^	20.67 ± 1.93^b^	2.59 ± 0.60 ^b^	260.89 ± 28.72 ^a^
S75	3	24.25	ND	407.73 ± 206.12^a^	11.02 ± 0.93^c^	ND	268.08 ± 139.77 ^a^

When the enzymatic activity was too low to be quantified, non-detected (ND) is indicated. Values in the same column with different letters are significantly different (*p* < 0.05)

Regarding the effect of V_R_, a similar behavior was observed for both enzymes. In general, when the V_R_ was increased from 0.33 to 1, the top phase recovery also increased. However, when the V_R_ was increased to 3, recovery decreased. Systems with a higher V_R_, have higher concentrations of ethanol. Therefore, this loss in activity could be associated with denaturation of the enzymes at higher ethanol concentrations. Previous studies have demonstrated that high concentrations of this alcohol reduce the activity of both lipase and SP (Ooi et al. 2009; Amid et al. 2012). Similar results were also observed by Simental-Martínez et al. (2014) when characterizing the partition of superoxide dismutase in ethanol–potassium phosphate ATPS.

**Fig. 4:**
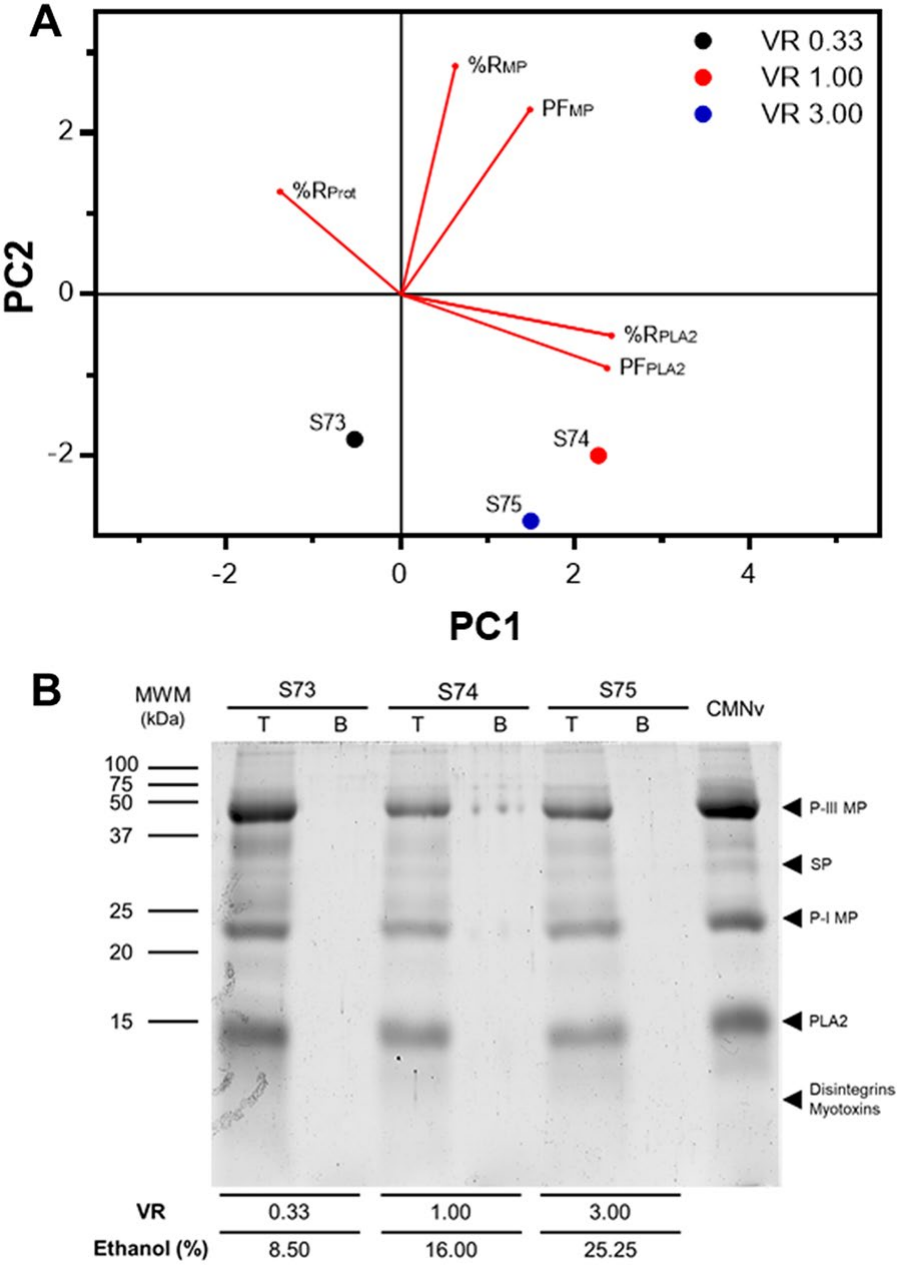
Recovery of MPs and PLA_2_ in ethanol–salt systems. **A** Principal component analysis (PCA) analysis of the recovery and purification of MPs and PLA_2_ in ethanol–salt systems at different volume ratio (V_R_) (0.33, 1 and 3). **B** SDS-PAGE of top and bottom phases (T and B, respectively) of ethanol–salt systems, 15 μg of *C. m. nigrescens* venom (CMNv) and molecular weight marker (MWM)

The recovery of MPs and PLA_2_ in ethanol–salt systems was summarized using PCA (Fig. 4A). It was evident that PLA_2_ activity was enhanced in this type of system. In fact, the pattern was similar to the one observed in PEG–salt systems with NaCl (see Fig. 2A), in which systems distribution was driven towards PLA_2_ PF and %R and an absence of MPs activity. Regarding electrophoretic analysis, no bands were observed in the bottom phases of these systems on SDS-PAGE (Fig. 4B). However, in systems S74 and S75, the intensity of MPs bands decreased in comparison to *C. m. nigrescens* venom (Additional file 1: Figure S1D). This behavior could be attributed to MPs migration to the interphase, which was probably occasioned by the high ethanol concentration in the systems. A similar behavior has been suggested for other type of molecules in alcohol–salt systems at increasing ethanol concentrations (Zhang et al. 2013).

## Conclusions

In this study, the recovery of MPs and PLA_2_ from *C. molossus nigrescens* venom using ATPS was investigated. After the evaluation of the effect of different system parameters, results showed that in PEG–potassium phosphate systems selective recovery of MPs and PLA_2_ was achieved. For recovery of PLA_2_, a system with PEG 400 g mol^−1^, V_R_ 1, TLL 25% w/w and pH 7.0 showed the best performance. In systems with PEG 400 g mol^−1^, V_R_ 1, TLL 15% w/w, pH 7.0 and NaCl concentrations of 1 and 3% w/w, selective recovery of P-III MP in the bottom phase was achieved; whereas a system with PEG 400 g mol^−1^, V_R_ 1, TLL 25% w/w and pH 8.5 allowed to recover different MPs subtypes from the venom in the bottom phase. These systems were selected based on the best recovery and purification performance of either MPs or PLA_2_ determined by PCA, as well as electrophoretic analysis of both phases. Ethanol–salt systems at three different V_R_ (0.33, 1 and 3) were also tested, however failed to differentially partition PLA_2_ and MPs. As a recommendation for future studies, the partition of PLA_2_ and MPs could be evaluated using ethanol–salt systems with lower ethanol concentrations. Also, alcohol–salt systems of a milder solvent, such as propanol could be tested.

The application of ATPS could potentially reduce the number of chromatographic steps usually required to purify snake venoms, a complex mixture of proteins, making this process simpler and cheaper. Once that the enzymes are separated, they may be recovered from the bottom phase through several methods including ultrafiltration, dialysis and the use of desalting columns. After enzyme recovery, the remaining salts in the bottom phase can be precipitated and used for a new extraction cycle. In addition, if higher enzyme purity is required, a chromatographic step may be included. The use of ATPS could potentially aid biochemical, biological and proteomic analyses of snake venoms and other complex toxin fluids.

### Supplementary Information

**Additional file 1: Table S1.** Composition of PEG-potassium phosphate systems used in this study. **Table S2.** Composition of ethanol-potassium phosphate systems used in this study.

**Additional file 2: Figure S1.** Densitometric analysis of MPs and PLA2 bands from SDS-PAGE ATPS systems. A) densitometric analysis of the selected PEG-potassium phosphate systems, B) PEG-potassium phosphate systems at different NaCl concentrations, C) PEG-potassium phosphate systems at pH values and D) ethanol-salt systems. The 47 kDa band correspond to P-III MPs, 24 to P-I MPs and the 14 kDa to PLA2..

## Data Availability

The datasets supporting the conclusions of this article are included within the article and its additional files.

## Abbreviations

ATPS: Aqueous two-phase systems
CMNv: *Crotalus molossus nigrescens* Venom
MP: Metalloproteases
PBS: Phosphate buffered saline
PCA: Principal component analysis
PEG: Polyethylene glycol
pI: Isoelectric point
PLA_2_: Phospholipases A2
PF: Purification factor
NaCl: Sodium chloride
%R: Recovery percentage
TLL: Tie line length
V_R_: Volume ratio
