# Supplementary material for: Development of aqueous two-phase systems-based approaches for the selective recovery of metalloproteases and phospholipases A2 toxins from Crotalus molossus nigrescens venom
Source: Bioresour Bioprocess. 2021 Dec 28;8(1):136. doi: 10.1186/s40643-021-00487-y (PMC10992436; doi:10.1186/s40643-021-00487-y)
Supplement: Supplementary file 1 — Additional file 1: Table S1. Composition of PEG-potassium phosphate systems used in this study. Table S2. Composition of ethanol-potassium phosphate systems used in this study. [file 40643_2021_487_MOESM1_ESM.docx]

Development of aqueous two-phase systems-based approaches for the selective recovery of metalloproteases and phospholipases A2 toxins from *Crotalus molossus nigrescens* venom

**Table S1.** Composition of PEG-potassium phosphate systems used in this study.

| **PEG molecular weight (g mol^-1^)** | **V_R_** | **TLL (% w/w)** | **PEG (% w/w)** | **Potassium phosphate (% w/w)** |
| --- | --- | --- | --- | --- |
| 400 | 0.33 | 15 | 9.7 | 22.3 |
|  |  | 25 | 10.2 | 23.0 |
|  |  | 35 | 10.0 | 27.0 |
|  |  | 45 | 10.0 | 29.0 |
|  | 1 | 15 | 16.5 | 16.0 |
|  |  | 25 | 17.2 | 15.5 |
|  |  | 35 | 21.0 | 16.9 |
|  |  | 45 | 22.0 | 17.0 |
|  | 3 | 15 | 17.5 | 15.0 |
|  |  | 25 | 19.5 | 13.7 |
|  |  | 35 | 22.3 | 13.0 |
|  |  | 45 | 27.0 | 12.5 |
| 1000 | 0.33 | 15 | 8.5 | 16.9 |
|  |  | 25 | 8.0 | 18.2 |
|  |  | 35 | 8.5 | 21.0 |
|  |  | 45 | 9.5 | 24.5 |
|  | 1 | 15 | 12.0 | 14.6 |
|  |  | 25 | 13.3 | 15.0 |
|  |  | 35 | 16.0 | 16.0 |
|  |  | 45 | 19.2 | 17.1 |
|  | 3 | 15 | 20.0 | 9.5 |
|  |  | 25 | 18.0 | 11.8 |
|  |  | 35 | 25.0 | 9.0 |
|  |  | 45 | 29.0 | 9.5 |
| 3350 | 0.33 | 15 | 6.0 | 13.0 |
|  |  | 25 | 7.3 | 14.0 |
|  |  | 35 | 7.3 | 18.2 |
|  |  | 45 | 9.2 | 21.0 |
|  | 1 | 15 | 19.2 | 17.1 |
|  |  | 25 | 10.0 | 10.8 |
|  |  | 35 | 15.0 | 13.2 |
|  |  | 45 | 18.8 | 15.0 |
|  | 3 | 15 | 14.0 | 8.5 |
|  |  | 25 | 19.0 | 7.5 |
|  |  | 35 | 26.0 | 6.5 |
|  |  | 45 | 30.0 | 8.0 |

**Table S2.** Composition of ethanol-potassium phosphate systems used in this study.

| **TLL (% w/w)** | **V_R_** | **Ethanol (% w/w)** | **Potassium phosphate (% w/w)** |
| --- | --- | --- | --- |
| 40 | 0.33 | 8.5 | 30.5 |
|  | 1 | 16.0 | 23.0 |
|  | 3 | 24.25 | 14.5 |
