# Supplementary material for: Development of aqueous two-phase systems-based approaches for the selective recovery of metalloproteases and phospholipases A2 toxins from Crotalus molossus nigrescens venom
Source: Bioresour Bioprocess. 2021 Dec 28;8(1):136. doi: 10.1186/s40643-021-00487-y (PMC10992436; doi:10.1186/s40643-021-00487-y)
Supplement: Supplementary file 2 — Additional file 2: Figure S1. Densitometric analysis of MPs and PLA2 bands from SDS-PAGE ATPS systems. A) densitometric analysis of the selected PEG-potassium phosphate systems, B) PEG-potassium phosphate systems at different NaCl concentrations, C) PEG-potassium phosphate systems at pH values and D) ethanol-salt systems. The 47 kDa band correspond to P-III MPs, 24 to P-I MPs and the 14 kDa to PLA2.. [file 40643_2021_487_MOESM2_ESM.docx]

**Figure S1. Densitometric analysis of MPs and PLA_2_ bands from SDS-PAGE ATPS systems.** A) densitometric analysis of the selected PEG-potassium phosphate systems, B) PEG-potassium phosphate systems at different NaCl concentrations, C) PEG-potassium phosphate systems at pH values and D) ethanol-salt systems. The 47 kDa band correspond to P-III MPs, 24 to P-I MPs and the 14 kDa to PLA_2_.
